# Simultaneous enterovirus EV-D68 and CVA6 infections causing acute respiratory distress syndrome and hand, foot and mouth disease

**DOI:** 10.1186/s12985-021-01560-w

**Published:** 2021-04-30

**Authors:** Ivanildo Pedro de Sousa, Heloísa Ihle Giamberardino, Sonia Mara Raboni, Maria Carmo Debur, Maria de Lourdes Aguiar Oliveira, Fernanda Marcicano Burlandy, Edson Elias da Silva

**Affiliations:** 1 Laboratório de Enterovírus, Instituto Oswaldo Cruz, Fundação Oswaldo Cruz, Rio de Janeiro, Brazil; 2 Divisão de Epidemiologia, Hospital Pequeno Príncipe, Curitiba, Brazil; 3 Laboratório de Virologia, Universidade Federal Do Paraná, Curitiba, Brazil; 4 Laboratório de Saúde Pública, Secretaria de Saúde do Estado do Paraná, Curitiba, Brazil; 5 Laboratório de Vírus Respiratórios e do Sarampo, Instituto Oswaldo Cruz, Fundação Oswaldo Cruz, Rio de Janeiro, Brazil

**Keywords:** Enterovirus, Hand, foot and mouth disease, Acute respiratory distress syndrome, EV-D68, CVA6

## Abstract

**Background:**

Although most enterovirus (EV) infections can be asymptomatic, these viral agents can cause serious conditions associated with central nervous system, respiratory disease and uncommon manifestations of hand, foot and mouth disease (HFMD). EV-coinfections have been rarely reported with development of complications and severe clinical outcome. An atypical case of a child presenting HFMD and severe acute respiratory syndrome, co-infected with EV-D68 and CVA6, is reported herein.

**Case presentation:**

A 3-year-old boy was admitted in the emergency department unit showing fever, abdominal pain and tachycardia. Twenty-four hours after hospitalization the child developed severe clinical symptoms associated with HFMD and was discharged after recovery. Two days later, the child was readmitted with fever, cough and respiratory distress. RT-PCR and Sanger sequencing confirmed positivity for EV-D68 and CVA6 in oro and nasopharynges swabs and vesicles fluid, respectively. Phylogenetic analysis based on VP1 gene sequences suggested that CVA6 was closely related with HFMD viruses circulating in Turkey, while EV-D68 was genetically related to a Chinese strain.

**Conclusions:**

To the best of our knowledge, this case is the first report of a double infection caused by CVA6 and EV-D68, which shed light on the pathogenesis of enterovirus infections. Further studies must be conducted to ascertain the role and clinical significance of EV co-infections, as well as a potential synergistic pathway between these viruses.

## Background

Enteroviruses (EVs) (genus *Enterovirus*, family *Picornaviridae*) are small, non-enveloped RNA viruses classified into fifteen species, seven of which (EV A-D and HRV A-C) are able to cause human infections [1, 2]. They are known to cause a broad spectrum of clinical manifestations, such as hand, foot and mouth disease (HFMD), CNS syndromes and severe acute respiratory syndrome (SARI) [3]. HFMD is a common childhood disease, generally self-limited and characterized by a benign febrile exanthema. Despite of this, a small proportion of patients may experience more severe forms of the disease. SARI is characterized by respiratory distress in association with other influenza-like illness symptoms [4].

Worldwide, enteroviruses play an important role in morbidity and mortality. Currently, EV-D68, EV-A71, rhinoviruses, and some EV-B species are the most important enteroviruses associated with respiratory infections. Additionally, recent outbreaks associated with CVA6 (HFMD) and EV-D68 (SARI) raised concern due to relevant neurological and respiratory complications, observed in some of the cases [5, 6]. In the present study, we report an unusual case of enterovirus co-infection in a patient with suggestive symptoms of HFMD and SARI. To date, this case is the first report of a putative enterovirus double infection caused by CVA6 and EV-D68.

## Case presentation

A 3 three years old boy was admitted into the emergency department unit (ED) from a pediatric hospital in Curitiba, PR, Brazil, showing fever (38 °C), abdominal pain and tachycardia. The patient presented a history of chronic renal disease and myelomeningocele (previously corrected by surgery). Twenty-four hours after hospitalization, erythematous and pruritic lesions arose on the palms and soles of the feet, perioral region and ears, which further evolved to vesicles in hands, feet, mouth, and ears. Four days after the admission, the symptoms improved and the patient was discharged. Two days later, the child returned with fever (39 °C), cough, respiratory distress with O_2_ saturation of 85–88%, being readmitted due to dyspnea. The child was referred to the Pediatric Intensive Care Unit (PICU) and submitted to mechanical ventilation. Skin examination revealed vesicles and crusts on hands, face, and ears. In the following day, renal function complications led patient to be submitted to hemodialysis. The patient was sedated, clinically managed, treated with antibiotics (piperacillin tazobactam) and, subsequently, improved O_2_ saturation. However, blood pressure (BP) remained unstable—fluctuating between periods of hypo- and hypertension (BP peaks > 200/110), stabilized after the onset of peritoneal dialysis. The echocardiogram showed major systolic and diastolic dysfunctions in the left and the right ventricles (LV and RV) and a major pulmonary arterial hypertension, suggestive of acute myocarditis. Chest X-ray showed reduced transparency at the base of the right lung, and absence of pulmonary consolidation. Blood cultures were performed with negative results. After stabilization of clinical conditions, mechanical ventilation was halted and the child was extubated, after 14 days of hospitalization. Subsequent echocardiography showed improvement of RV systolic function and normal pulmonary arterial pressure. He was discharged from the PICU after 16 days and was maintained in oxygen therapy (O_2_ saturation of 98%). After complete recovery, the patient was discharged. The chronological order of events is shown in the Table 1.

**Table 1:** Summary of the chronological order of events from admission to discharge

Date	Event
Initial presentation	Symptoms: fever (38 °C), abdominal pain and tachycardia
Days 2–5 post-initial presentation	Erythematous and pruritic lesions arose on the palms and soles of the feet, perioral region and ears, which further evolved to vesicles in hands, feet, mouth, and ears
Days 6–14 post-initial presentation (second admission)	Fever (39 °C), cough, respiratory distress (O_2_ saturation of 85–88%), vesicles and crusts on hands, face, and ears, blood pressure unstable, acute myocarditis
Days 15–16 post-initial presentation	Improvement of systolic function and arterial pressure. The child was discharged from the PICU and maintained in oxygen therapy (O_2_ saturation of 98%)
Days 20 post-initial presentation	After complete recovery, the patient was discharged

Oro and nasopharynges swabs and vesicles fluid were collected and sent to the Public Health Laboratory of Paraná (LACEN/PR) for the identification of the causative agent. Clinical samples were also referred to the Enterovirus Laboratory (Oswaldo Cruz Institute/Fiocruz, Rio de Janeiro, Brazil) for further investigation, as part of the National Enteroviruses Surveillance Program. Samples were inoculated into RD (human rhabdomyosarcoma) and HEp-2C (human epidermoid carcinoma) cell lines, which were then incubated at 37 °C or 33 °C and examined daily for the presence of a cytopathic effect (CPE). Both cell cultures failed to yield CPE. Viral RNA was extracted (Viral Nucleic Acid Extraction Kit—QIAmp-Qiagen) directly from the clinical specimens and initially subjected to a broad-reactive real time RT-PCR for human enteroviruses as previously described [7]. EV-positive samples were then subjected to a semi-nested RT-PCR for amplification of partial VP1 gene as described [8]_._ EV-positive amplicons (~ 350 nt.) were sequenced by the Sanger method using a BigDye Terminator v3.1 Cycle Sequencing Kit (Applied Biosystems). Nucleotide sequences obtained were compared with those available at GenBank, which confirmed the presence of EV-D68 and CVA6 in nasopharyngeal and blisters swabs, respectively. A multiplex real time PCR-based assay was also performed for the simultaneous detection of respiratory viruses (influenza A, influenza B, syncytial respiratory virus, adenovirus, metapneumovirus, bocavirus, parainfluenza 1, 2 and 3, coronaviruses OC43, HKU1, NL63 and 229E) by using a commercial assay (Seeplex RV15 ACE detection kit, Seegene, Korea), but yielded non-detectable results, which excludes these pathogens as those responsible for the SARI presentation.

**Fig. 1:**
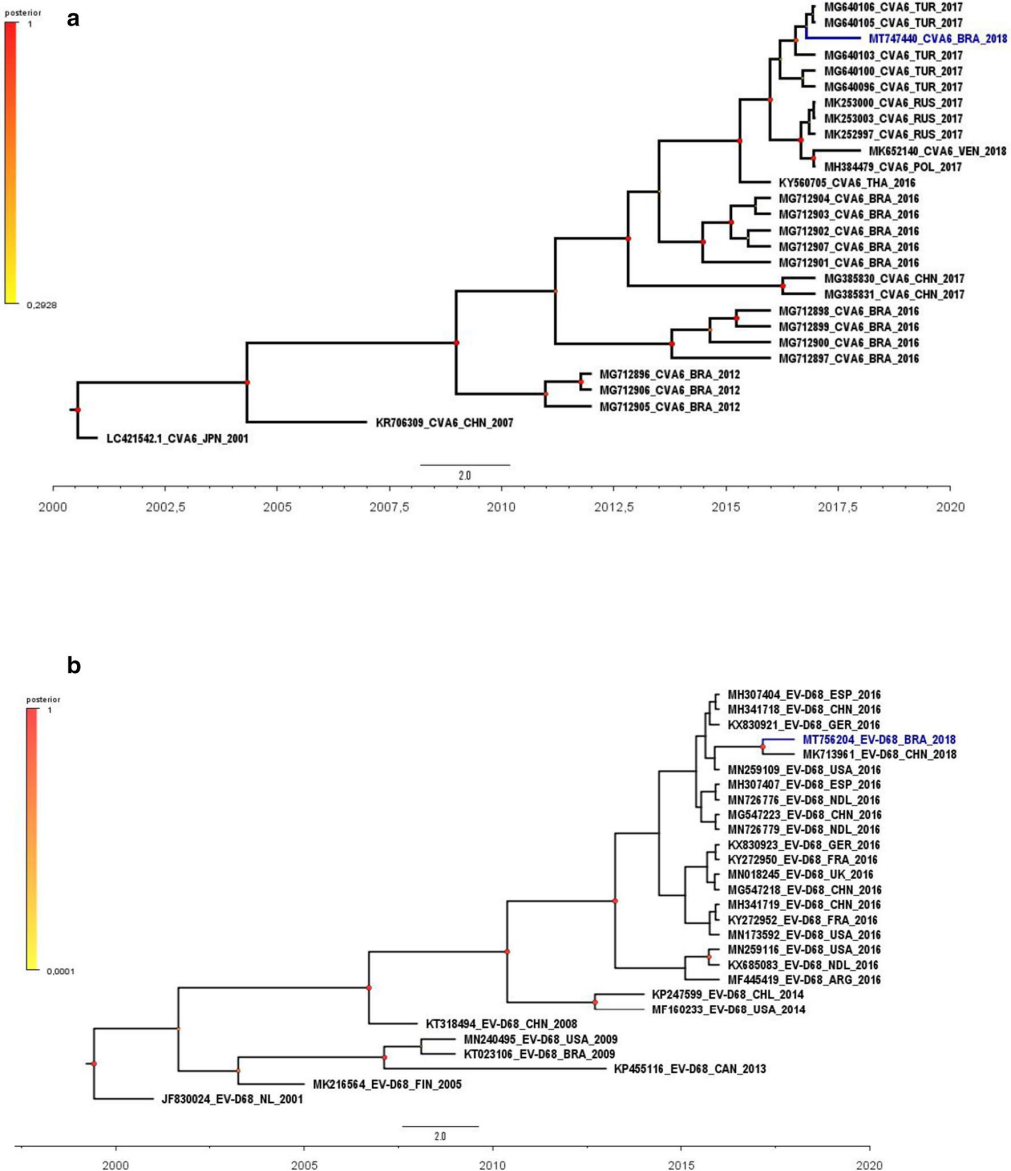
Phylogenetic analysis based on CVA6 (218 bp) (**a**) and EV-D68 (205 bp) (**b**) partial VP1 sequences. Maximum clade credibility (MCC) tree based on the Bayesian analysis of the VP1 nucleotide sequences and their closely related sequences. The samples of this study are represented by blue color. Branches are in time scale (year). Posterior probabilities are shown as a color scale. The scale bar indicates years. The strain name, year of sampling and GenBank accession numbers are presented

The EV RT-PCR results were confirmed by phylogenetic analysis. Representative EV-D68 and CVA6 sequences were downloaded from Genbank (https://www.ncbi.nlm.nih.gov/nucleotide/) and added to our datasets. For phylogenetic reconstruction, sequences were aligned using Muscle [9] available in the Mega 7.0 package [10] and the best nucleotide substitution model was determined by using JModeltest, version 2.1.4 [11]. The temporal structure of the dataset was verified using TempEst v. 1.5.1 [12]. Afterwards, phylogenetic trees were reconstructed by a Bayesian Markov Chain Monte Carlo (MCMC) method, accessible in the BEAST software package, version 1.10 [13, 14]. Time calibration was set based on the year of sample collection, and the general time reversible (GTR) with gamma-distributed rates and invariant sites was employed as the nucleotide substitution model. Beast runs were carried out using the uncorrelated lognormal relaxed molecular clock model and a time-aware Gaussian Markov Random Field (GMRF) Bayesian skyride coalescent tree prior [15, 16]. The length of MCMC chains were established as 40 million, sampled every 40,000 steps. Trace files generated through Bayesian phylogenetic inference were visualized and analyzed in Tracer version 1.7 [17]. Convergence of parameters was considered in the presence of effective sample size (ESS) values exceeding 200. The target Maximum Clade Credibility (MCC) tree was summarized by TreeAnnotator 1.8.4, with a burn-in corresponding to 10% of states. MCC trees were visualized and edited in FigTree, version 1.4.3 (http://tree.bio.ed.ac.uk/software/figtree/). Beast runs were performed in CIPRES Science Gateway [18]. Viral sequences were deposited at GenBank (NCBI), under the accession numbers MT747440 (CVA6) and MT756204 (EVD-68). The Bayesian phylogenetic trees of partial VP1 gene sequences are shown in Fig. 1. The CVA6 sequence showed a clear phylogenetic relationship with HFMD viruses circulating in Turkey in 2017 (Fig. 1a). EV-D68 sequence grouped into a contemporary clade, comprising global representatives from 2016 to 2018, and exhibited a robust phylogenetic relatedness with a Chinese sequence (MK713961) (Fig. 1b). Neither EV-D68 nor CVA6 were genetically related to viruses known to be circulating in Brazil.

## Discussion and conclusions

Worldwide, CVA6 emerged as an important causative agent of HFMD outbreaks, sometimes producing atypical clinical manifestations [19–22]. The main features of these atypical infections consist of a widespread distribution of rash and blisters [22], not frequently observed in traditional HFMD cases. In this study, the patient presented extensive clinical symptoms of HFMD, with a wider range of rash and skin lesions.

Until 2010, EV-D68 infections were sporadically reported worldwide. However, in recent years, an increasing number of cases with neurological symptoms, compatible with acute flaccid myelitis, and severe acute respiratory infections have been described [23]. EV-D68 was firstly detected in Brazil in 2009 [24]. Since then, the virus had not been identified in SARI or neurological patients, until a recent report in which this virus was detected in respiratory samples collected during an extensive population-based laboratory surveillance in southern Brazil [25]. Additionally, co-infection by distinct enteroviruses seems to enhance the pathogenic effect of the disease. Indeed, recent reports have described that these co-infections can lead to synergies on the pathogenic mechanisms [26, 27]. Furthermore, even though the occurrence of infection by a single EV-type can result in severe clinical syndromes, the EV-coinfection can lead to increased possibility of developing complications and severe clinical aspects of disease [27]. Interestingly, previous work have demonstrated that the co-circulation of a high number of different NPEV types can favors recombination events as well as the emergence of the atypical clinical presentations and the severity of disease [28–32]. Additionally, the cross-neutralizing antibodies against different EV-types is not commonly observed, mainly in children ≤ 6 years [32].

Moreover, these enteroviruses present a high genetic diversity, a consequence of cumulative mutations and/or recombination events. Indeed, during viral co-infections a large number of genetic variants can coexist in the host, leading to emergence of more pathogenic species, which can change the dynamic of infections [33, 34]. Additionally, the synergistic effect of these infections should not be disregarded [33]. Thus, monitoring EV co-infections is critical to understand atypical clinical cases, with a range of disease severity. We are uncertain whether myocarditis observed in the current case was a consequence of the described EV coinfection. Further studies must be conducted to ascertain the putative synergic mechanisms between viruses and the resulting clinical impacts.

The typing of non-polio enteroviruses is conventionally depended on virus isolation in cell culture. However, the major drawback is that enteroviruses are frequently not identified due to the fact that some of them replicate poorly in culture [35]. In this study, the clinical specimens obtained from the patient failed to yield CPE. Even though EV-D68 has been shown to replicate in special condition (33 °C) than normally applied for other EV, we also were unable to isolate it in this condition. Additionally, other studies have pointed for a lower efficiency of isolation [19] and that enterovirus isolation rate can suffer fluctuation depending on the cell line used [36, 37]. However, other factors could explain the absence of CPE, such as prolonged storage of clinical specimens and delays in their transportation to the laboratory, alongside the transport temperature [38].

The main limitations of the present report consist of the small VP1 RT-PCR fragment, which possibly precludes a better exploration of phylogenetic relationships. In this way, outcomes must be cautiously considered, in the light of this limitation. In order to solve this limitation next generation sequencing (NGS) techniques provides solution for fast full-genome sequencing with high efficiency and deeper of analysis to enable identification of multiple viruses from the same specimen [39]. Likewise, the challenging access to the patient´s records and additional information was a key restraint.

The current study highlights the capacity of EV surveillance in detecting emergent EV-types with potential to cause severe disease, as well as their possible co-infections. The continuous monitoring of circulating viruses is pivotal to ameliorate the current public health strategies. Lastly, the high frequency of viral intra- and intergroup genetic recombination reinforces the value of virological surveillance to timely identify novel recombinant viruses, co-infections and other events, which contribute to disease severity and burden.

## Data Availability

The data analyzed during the current study are available from the corresponding author on reasonable request.

## Abbreviations

CV: Coxsackievirus
CNS: Central nervous system
EV: Enterovirus
E: Echovirus
HFMD: Hand, foot and mouth disease
SARI: Severe acute respiratory syndrome
